# Update of Assessment of Survival in Head and Neck Cancer after Regional Recurrence

**DOI:** 10.1155/2012/154303

**Published:** 2012-10-10

**Authors:** Ali Amar, Helma Maria Chedid, Abrão Rapoport, Rogério Aparecido Dedivitis, Claudio Roberto Cernea, Lenine Garcia Brandão, Otavio Alberto Curioni

**Affiliations:** ^1^Department of Head and Neck Surgery and Otorhinolaryngology of the Heliópolis Hospital, São Paulo, Brazil; ^2^Department of Head and Neck Surgery, Hospital das Clínicas, São Paulo School of Medicine, University of São Paulo, São Paulo, Brazil

## Abstract

*Objective*. To evaluate site of regional recurrence in patients with squamous cell carcinoma of upper aerodigestive tract after neck dissection and the results of salvage treatment. *Methods*. 95 patients with regional recurrence as the first manifestation of relapse were selected between 943 patients who underwent neck dissection. We evaluated level and side of recurrence, as well disease control after salvage treatment. *Results*. Level II was the most frequent site of recurrence. Salvage treatment was performed in 51% of ipsilateral and in 75% of contralateral (nondissected neck) recurrences. Control of the disease 12 months after salvage surgery was 25% in the ipsilateral and 37% in contralateral recurrences. *Conclusions*. Cervical recurrences occur predominantly in level II. Relapse in level I is frequent only in oral cavity tumors and relapse in level V is rare. The neck recurrence carries a poor prognosis even among patients who underwent retreatment with curative intent.

## 1. Introduction

Treatment of head and neck cancer varies according to the primary site, tumor stage, patient treatment preference, and practitioner's expertise. Neck lymph nodes approach depends on the primary site and extent of disease [1]. The efficacy of elective neck dissection has been demonstrated due to predictable pathways of lymphatic drainage and metastasis [2].

Although neck dissection is effective to control regional disease, the presence of lymph node metastases is associated with significant decrease in survival rate, since the cervical metastatic disease is also associated with increase of local and distant recurrences. Recurrence in the neck offers a higher risk of mortality as opposed to local failure [1]. Failure in the neck after definitive neck treatment offers poorer prognosis than local recurrence [3]. Recurrence after multimodal treatment, in turn, presents poor prognosis with long-term survival of less than 5% [4–7]. Recurrence in the neck can result from observation of the neck, inadequate regional treatment, or missed metastasis at the time of initial approach.

Postoperative radiotherapy has been recommended in order to reduce the incidence of regional recurrence, especially in the presence of multiple metastatic lymph nodes and capsular rupture, assuming that it is better to treat microscopic than extensive disease. The adjuvant treatment considers the likelihood of residual disease and the difficulty to perform another surgery in a previous operated area. The unsatisfactory results of salvage treatment, reflecting aggressive tumors, open space for the development of new therapeutic strategies. 

The aim of this study was to evaluate site of regional recurrence in patients with squamous cell carcinoma of upper aerodigestive tract after neck dissection and the results of salvage treatment.

## 2. Methods

The records of 943 patients with squamous cell carcinoma of the mouth, oropharynx, hypopharynx, and larynx were evaluated. All patients underwent surgical treatment of primary tumor and neck dissection at the Department of Head and Neck Surgery of Heliopolis Hospital.

Neck recurrence was diagnosed in 124 patients, of whom 29 were excluded because local recurrence or second tumor was associated with regional recurrence, leaving 95 patients with neck recurrence as the first manifestation of relapse.

Among the 95 patients, 87 were male and eight were female. The primary site and stage are shown in Table 1. Unilateral radical neck dissection was performed in 70 patients, radical bilateral in 20, selective unilateral in four, and radical plus selective contralateral in one patient. The pathological examination of these specimens was pN0 in 27 and pN+ in 68 patients. Postoperative radiotherapy was performed in 44 patients, including the primary site and both sides of the neck with a mean dose of 53 Gy.   We evaluated the site of recurrence (five-level nodes and laterality) in front of the primary site and postoperative radiotherapy. Relapses in patients who underwent bilateral neck dissection were included among ipsilateral failures. The involvement of different lymph node levels were expressed in number of patients with involvement at that level, considering recurrences at multiple levels. We evaluated the salvage treatment only in unilateral recurrence, taking into account the previous treatment (with or without radiotherapy) and the side of recurrence.

The salvage surgery in a previous dissected neck was a lymphadenectomy and a radical neck dissection for contralateral (non-dissected neck) recurrences. Control of the disease in the neck was evaluated by Kaplan-Meier method. A successful treatment was defined as disease control for at least 12 months after the salvage.

## 3. Results

The location of the recurrence was the dissected neck in 49 patients, contralateral (non-dissected) neck in 36, and five recurrences on both sides, and in five patients the side of relapse was not determined. The level of recurrence was not recorded in two patients. The laterality and level of recurrences are shown in Table 2. Three patients who underwent radical neck dissection had ipsilateral recurrences outside the operative field; one patient developed recurrence in the parotid gland, one in the retropharyngeal nodes, and one in the occipital region. One patient undergoing supraomohyoid neck dissection had recurrence in level IV. The remaining ipsilateral recurrences, even in cases of selective neck dissection occurred in the operative field. The median time between initial treatment and neck recurrences was 210 days. Ipsilateral recurrences occurred in level II in 57%, accounting for 61% (8/13) of recurrences in pN0 and 56% (23/41) of recurrences in pN+ patients. Regarding postoperative radiotherapy, ipsilateral recurrences at level II occurred in 67% (21/31) of nonirradiated and in 43% (10/23) of irradiated necks. The level of recurrence for each primary site in the previous dissected neck is shown in Table 3.

Among patients with unilateral recurrence, the salvage treatment was performed in 52 patients—Tables 4, 5, and 6. It was performed in 51% of ipsilateral and 75% of contralateral recurrences. In 33 patients the treatment was chemotherapy or palliative care. There were three postoperative deaths. Among treated patients, four were asymptomatic at the time of last visit and were lost to followup before 12 months posttreatment. The control of disease after 12 months was 17%, being 12% for ipsilateral and 25% for contralateral recurrences. Considering only treated patients and excluding those lost to followup, the control rises to 31%, 25%, and 37%, respectively.

**Figure 1:**
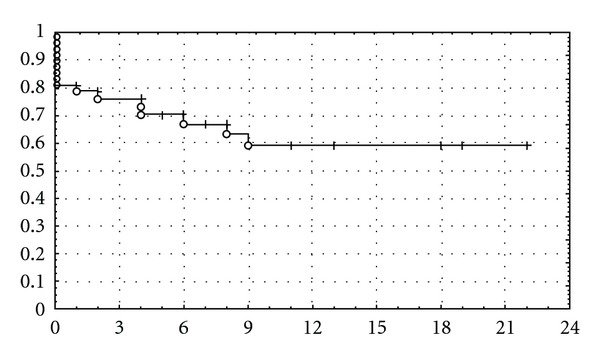


The neck control after 12 months was 59% for patients with recurrent unilateral neck who underwent salvage treatment, 43% for ipsilateral, and 65% for contralateral recurrences (Figure 1). All neck recurrences after salvage therapy were diagnosed in the first year after salvage treatment. The data correspond to 47 patients that contained this information in their records.

The distant metastases were diagnosed in 13 patients with regional recurrence, and in eight of them only after the salvage treatment. After salvage treatment nine patients had local recurrence between the 2nd and 10th months and two patients developed a second primary tumor after 6 months and 5 years.

## 4. Discussion

The location of cervical recurrence is similar to the usual distribution of metastases, usually affecting level II. Relapse in level I is frequent only in tumors of the mouth and relapse in level V is rare. Relapses often occur within operative field, even in selective neck dissection, emphasizing the therapeutic value of this procedure [8]. Recurrences after radiotherapy usually occur in the area of previous involved lymph nodes, despite the use of higher doses [9]. In addition to the barrier effect, the first station of lymph nodes may have favorable conditions for metastatic development [10]. A recurrence at level II on a previously dissected neck is generally not subject to salvage, especially if postoperative radiotherapy was done. The contralateral recurrences reopen the discussion about elective or therapeutic neck dissection. Sometimes, subclinical cervical metastases show explosive growth after removal of the primary tumor. The recurrences in undissected neck can be treated very often, but despite the neck control overall survival is poor [11].

The elimination of level V and IIb lymph nodes dissection can lower the incidence of spinal nerve injuries, preserve the roots of the deep cervical plexus, and reduce surgical time [12]. Level IIb lymph nodes contained in the submuscular recess are the lymph nodes lying over the fascia of the *splenius capitis* and *levator scapulae*, above the spinal accessory nerve, posterolaterally bordered by the sternocleidomastoid muscle and superiorly by the skull base [13]. In general, the risk of metastasis at level IIb is low. However, this is statistically significant in the case of N+ (33.3%) (*P* < 0.05) or when level IIa is involved (*P* < 0.05). The percentage of metastases at level IIb in oral cavity cancer N0 neck looks as if it would appear to range from 0% to 22%. Thus, the indication for dissection that level remains controversial. For other site (larynx, for example) with N0 neck, there is general agreement that suggests leaving the cervical IIb lymph nodes undissected [12].

The rationale for selective neck dissection is based on the predictability pattern of lymph node metastasis. Of the 5 nodal levels, only 3 are typically considered most at risk of being pathologically involved. As a result, proponents of selective neck dissection believe that a targeted removal of only the nodal levels that are predicted to be most at risk of metastasis is adequate to achieve regional control [14].

Since a patient is a candidate for salvage surgery, the extent of surgical resection should be determined by the location and extent of the original tumor. Recurrent disease is often understaged by clinical examination and imaging studies [15]. Experimental studies show that active disease may contribute to the quiescence of metastatic foci, and treatment of metastasis may promote the growth of subclinical disease in other sites, both related to angiogenic modulation [16–18].

The incidence of distant metastases was probably underestimated among inoperable patients, not submitted to additional investigation. Recurrences at the primary site and distant metastases are responsible for a significant number of failures after salvage treatment of the neck, but subclinical disease cannot be excluded in the neck in many cases that died without enough time to manifest recurrence. The PET-TC could be useful in this situation.

Localized therapy is insufficient in most of these patients and the success of treatment could be related to a technical failure in the initial treatment. Despite low rates of survival, neck control justifies the treatment. Since the recurrence attests the ineffectiveness of previous treatment, heroic measures are not justified with the same therapeutic modalities, when quality of life becomes mandatory.

The surgical salvage and survival rates are similar to those rates reported by other authors as well as reported after radiotherapy or radiochemotherapy [19, 20]. In a prospective randomized study of selective neck dissection versus observation for N0 neck of early tongue carcinoma, all 11 patients with isolated nodal recurrence had successful salvage [21]. On the other hand, in a retrospective review of 70 patients with N0 disease who underwent partial glossectomy for pT1/pT2 oral tongue cancer without any neck surgery, 20 patients developed isolated nodal failure after watchful waiting policy. For all patients with initial T1 disease, the nodal recurrence was controlled. However, patients with T2 tumors did not do well [22].

Salvage treatments can have complications, either because they are more invasive surgically or the tissue in the area of dissection has already been compromised by previous therapy. Surgery is often the treatment of choice for salvage neck therapy [1].

Considering the areas at risk for developing recurrence, postoperative radiotherapy may follow the same anatomical principles of selective dissection, especially after comprehensive neck dissection. The reverse rationale, that is, performing selective dissection following radiotherapy, is also effective in the management of neck metastases in selected cases [14].

## 5. Conclusions

Cervical recurrences occur predominantly in level II. Relapse in level I is frequent only in oral cavity tumors and relapse in level V is rare. The neck recurrence carries a poor prognosis even among patients who underwent retreatment with curative intent.

## Figures and Tables

**Table 1:** Distribution of patients according primary site and stage.

Primary site	Stage				Total
	I	II	III	IV
Oral cavity	0	4	11	14	29
Oropharynx	0	0	4	7	11
Hypopharynx	1	0	6	25	32
Larynx	0	0	13	8	21

Total	1	4	34	54	93

^∗^2 patients without staging information.

**Table 2:** Side and level of neck recurrences in 86 patients.

Recurrence	Ipsilateral	Contralateral (non-dissected)
Level I	6	4
Level II	31	20
Level III	7	12
Level IV	6	2
Level V	5	3

^∗^5 patients with bilateral recurrences and 5 patients with metastasis in two levels.

^∗∗^Patients without information or recurrences outside the five levels were excluded.

**Table 3:** Level of neck recurrences in the previous dissected neck (ipsilateral) for each primary site.

	Oral cavity *n* = 16	Oropharynx *n* = 8*	Hypopharynx *n* = 20	Larynx *n* = 10
Level I	4	1	1	0
Level II	7	5	11	8
Level III	2	1	4	0
Level IV	2	1	3	0
Level V	1	1	1	2

^∗^1 patient with recurrence in 2 levels.

**Table 4:** Treatment of unilateral neck recurrences.

Neck recurrence side	*N*	Treatment				Neck control
		Surgery	S + RT	RT	Palliative
Ipsilateral	49	6	7	12	24	10
Contralateral	36	12	10	5	9	20

Total	85	18	17	17	33	30

**Table 5:** Side and level of neck recurrences in 86 patients.

Recurrence	Ipsilateral	Contralateral
Level I	6	4
Level II	31	20
Level III	7	12
Level IV	6	2
Level V	5	3

^∗^5 patients with bilateral recurrences and 5 patients with metastasis in two levels.

**Table 6:** Level of neck recurrences for each primary site.

	Oral cavity	Oropharynx	Hypopharynx	Larynx
Level I	4	1	1	0
Level II	7	5	11	8
Level III	2	1	4	0
Level IV	2	1	3	0
Level V	1	1	1	2
